# Supplementary material for: Lasso-grafting of macrocyclic peptide pharmacophores yields multi-functional proteins
Source: Nat Commun. 2021 Mar 9;12:1543. doi: 10.1038/s41467-021-21875-0 (PMC7943567; doi:10.1038/s41467-021-21875-0)
Supplement: Supplementary file 1 — Supplementary Information [file 41467_2021_21875_MOESM1_ESM.pdf]

Supplementary Information

Supplementary Figures

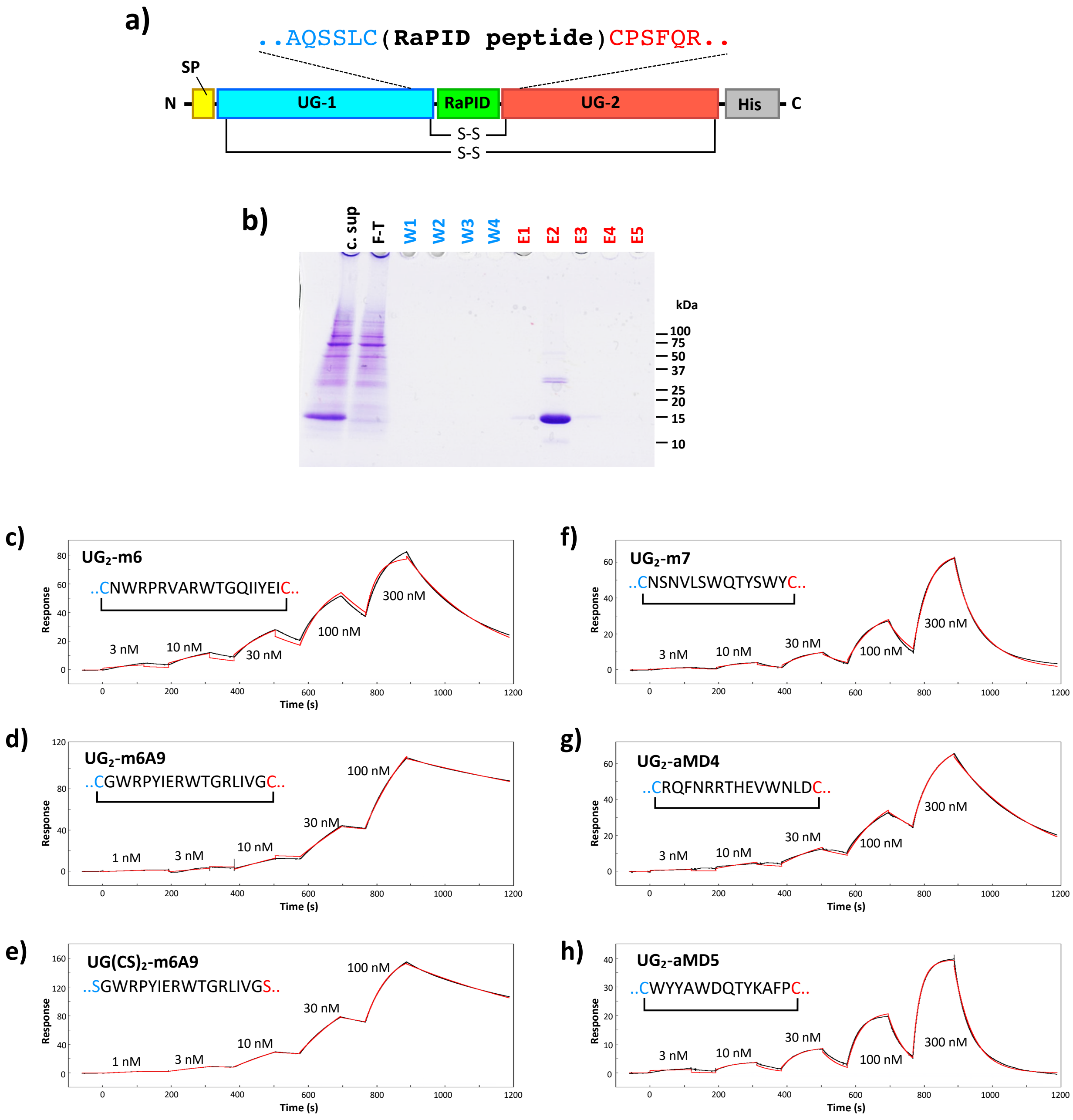

Supplementary Figure S1. Peptide grafting onto homodimeric uteroglobin. **a**, Construct design of peptide grafts onto UG. The construct is comprised of a signal peptide (SP), the first UG subunit (UG-1), the grafted RaPID-derived peptide, the second UG subunit (UG-2), and a $(His)_6$ tag. Disulfide bonding patterns are indicated by brackets. **b**, Purification of peptide-grafted UG tandem dimers using Ni-NTA-agarose chromatography. A representative nonreducing SDS-PAGE (15-25% gradient gel) is shown for samples taken during the purification of m6-grafted UG (UG2-m6). Samples include the original culture supernatant (c. sup), the flow-through fraction (F-T), TBS wash fractions (W1-W4), and 0.25 M imidazole elution fractions (E1-E5). **c-h**, Biacore sensorgrams from kinetic binding analyses of peptide-grafted UGs. Samples were serially diluted to the indicated concentrations and successively flowed over sensorchips with immobilized ectodomain fragments of PlxnB1 (**c-f**) or MET (**g, h**) in the “single-cycle kinetics” mode to derive kinetic rate constants shown in Fig. 1c. The actual amino acid sequences of the grafted peptide is shown below each sample name. Note that the peptide-flanking cysteines are mutated to serines in $UG(CS)_2$-m6A9 (**e**). The sensorgrams and the fitted curves are shown in black and red lines, respectively.

a)

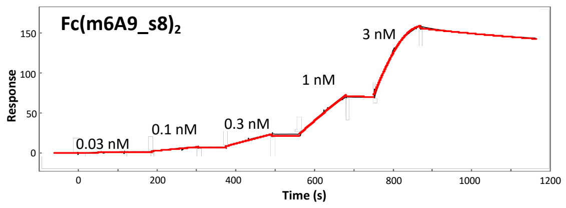

b)

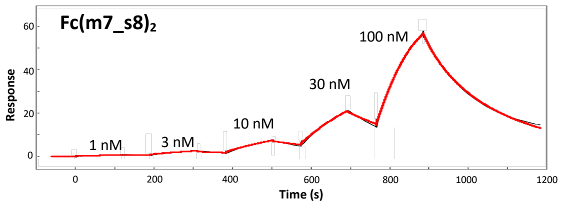

c)

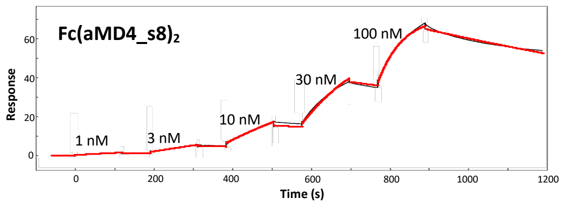

Supplementary Figure S2. Binding ability of peptide-grafted Fc proteins. **a-c**, Biacore sensorgrams for the kinetic binding analyses of the indicated peptide-grafted Fcs. Samples were serially diluted to the indicated concentrations and successively flowed over sensorchips with immobilized ectodomain fragments of PlxnB1 (**a**,**b**) or MET (**c**) in "single-cycle kinetics" mode to derive the kinetic rate constants shown in Fig. 1c. Note that the kinetic constants derived from these curve fitting do not reflect true 1:1 affinity due to the divalent nature of the Fc grafts.

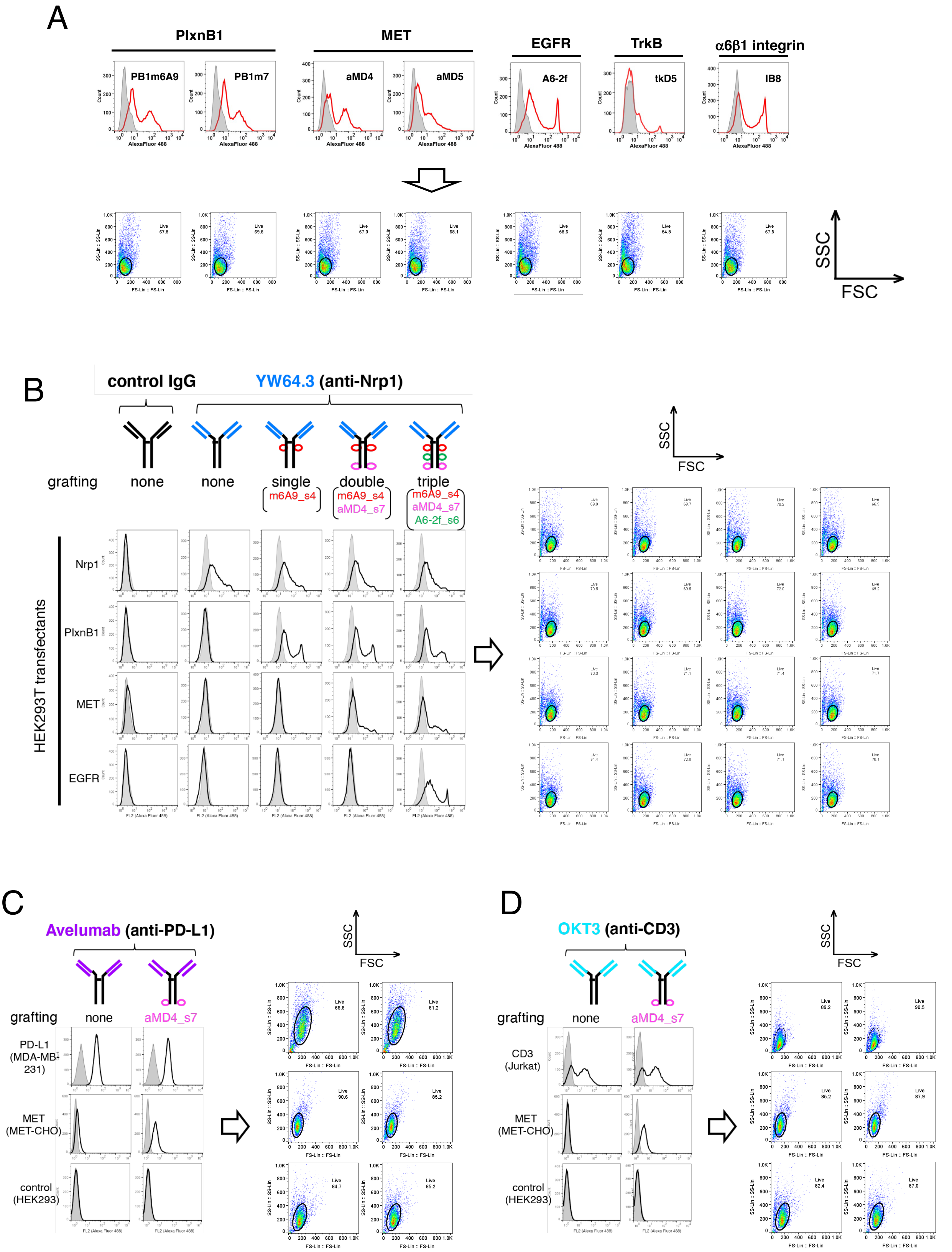

Supplementary Fig. S3. Flow cytometry gating strategies

Scatterplots showing live cell gating (black ovals) for each histogram panel in (A) peptide-grafted Fc binding to receptor-expressin 293T cells (relating to Fig.4) and (B-D) addbody binding to transfected HEK cell, MDA-MB-231 cells, MET-CHO cells, and Jurkat cells, respectively (relating to Fig.5a-c).

Supplementary Tables

Supplementary Table S1. Amino acid sequences of the peptides used in the grafting

name	target	original RaPID peptide[a]	sequence used for grafting[b]	Ref
PB1m6	Plexin B1	Ac-wRPRVARWTGQIIYC	WRPRVARWTGQIIY	(1)
PB1m6A9	Plexin B1	Ac-wRPYIERWTGRLIVC	WRPYIERWTGRLIV	(2)
PB1m7	Plexin B1	Ac-wNSNVLSWQTYSWYC	(C)NSNVLSWQTYSWY(C)	(1)
aMD4	MET	Ac-yRQFNRRTHEVWNLDC	YRQFNRRTHEVWNLD	(3)
aMD5	MET	Ac-yWYYAWDQTYKAFPC	YWYYAWDQTYKAFP	(3)
A6-2f	EGFR	Ac-BASFPEELDEWYVAYGC	(C)ASFPEELDEWYVAYG(C)	this study
tkD5	TrkB	Ac-yRQASPKFTFWSC	(C)YRQASPKFTFWS(C)	this study
IB8	integrin α6β1	Ac-YLVKRSGWLYTVDC	YLVKRSGWLYTVD	this study
PA tag	NZ-1 antibody	n/a	GVAMPGAEDDVV	(4)

[a] By convention, RaPID peptides are cyclized via the *N*-chloroacetyl group of the initiator amino acid and the sulfhydryl group of the terminal Cys. The small letter indicates *D*-amino acids, and B represents an unnatural amino acid *L*-boronophenylalanine.

[b] Flanking Cys residues (parenthesis) were added in some peptides to increase the binding capacity of the grafted proteins.

(1) Matsunaga et al., *Cell Chem. Biol.*, 23, 1341-1350 (2016)

(2) Bashiruddin et al., *Proc. Natl. Acad. Sci. U.S.A., 117, 31070-31077* (2020)

(3) Ito et al. *Nature Commun.*, 6, 6373 (2015)

(4) Fujii et al., *Protein Exp. Purif.*, 95, 240 (2014)

Supplementary Table S2. Scaffold proteins and insertion sites used for the peptide grafting

protein	length	SCOP domain(fold)	graft site	flanking sequence[a]
Fn10	93	Fn3(β-sandwich)	s1	...ISWDAP//VTVRYY..
			s2	...YAVTGR//SPASSK....
VHH	115	IgV(β-sandwich)	s1	...WVAGMS//RSSYED....
			s2	...YCNVNV//FEYWGQ...
SIRPα	118	IgV(β-sandwich)	s1	...ELIYNQ//KEGHFP...
			s2	...KFRKG//PDTEFK...
CEA	108	IgV(β-sandwich)	s1	...IIGYVI//QQATPG...
			s2	...TLHVIK//LVNEEA...
Fc	227	IgC(β-sandwich)	s1	...VVDVSH//DPEVKF...
			s2	...PREEQY//NSTYRV...
			s3	...SNKALP//PIEKTI...
			s4	...TISKAK//QPREPQ...
			s5	...PPVLDS//GSFFLY...
			s6	...RDELTK//QVSLTC...
			s7	...AVEWES//GQPENN...
			s8	...KSRWQQ//NVFSCS...
HSA	590	albumin(all α)	s1	...TCVADE//AENCDK...
			s2	...TECCQA//DKAACL...
			s3	...EKCCAA//DPHECY...
			s4	...EKCCKA//DKETCF...
hGH	185	4-helix cytokine (helical bundle)	s1	...RLED//SPRTGQ...
			s2	...DTNSHN//DALLKN...
RBP	201	lipocalin(β-barrel)	s1	...GRVRLL//NNWDVC...
			s2	...KYWGVA//SFLQKG...
ALP	489	alkaline phosphatase-like (α/β)	s1	...GPGYVL//GARPDV...
Cap (AAV2)	533	n/a	s1	...RTNTPS//TTQSRL...
			s2	...TNLQRG//QAATAD...
Cap (AAV1)	534	n/a	s1	...RTQNQS//AQNKDL...
			s2	...VNFQSS//DPATGD...

[a] RaPID-derived peptides (shown in Table S1) are inserted in the position indicated by "//", with up to 3 linker residues.

Supplementary Table S3. Primer sequences used for extension PCR

Relevant construct	Primer name	Strand	Sequence(5'- 3')
UG2-m6	UG-Fwd3	sense	TGGACCGGACAGATCATTACGAGATCTGCCCTTCCTTCCAACGCG
	UG-Rev3(-)	antisense	TCTCGCGACCCTAGGCCGCCAATTACACAGTGAGCTTTGGGCTATT
UG2-m6A9	mP69-Fwd24	sense	GTGGACAGGAAGGTTGATCGTGGGTTGCCCTTCCTTCCAACGCGTG
	mP69-Rev24(-)	antisense	CTCTCGATGTAAGGCCGCCAACCACACAGTGAGCTTTGGGCTATTT
UG(CS)2-m6A9	mP69-Fwd23	sense	GTGGACAGGAAGGTTGATCGTGGGTAGCCCTTCCTTCCAACGC
	mP69-Rev23(-)	antisense	CTCTCGATGTAAGGCCGCCAACCGGACAGTGAGCTTTGGGCTA
UG2-m7	P7ds-Fwd10	sense	GGCAAACTTACAGCTGGTATTGCGGTAGCCCTTCCTTCC
	P7ds-Rev10(-)	antisense	AGGACAGCACGTTAGAATTGCAACCGGACAGTGAGCTTTG
UG2-aMD4	MD4-Fwd21	sense	CACGAGGTGTGGAACTTGGATTGCGGTAGCCCTTCCTTCC
	MD4-Rev21(-)	antisense	CGTACGCCTGTTGAACTGCCGGCAACCGGACAGTGAGCTTTG
UG2-aMD5	MD5-Fwd13	sense	AGACCTACAAAGCCTTTCCGTGCGGTAGCCCTTCCTTCC
	MD5-Rev13(-)	antisense	GATCCCAAGCATAGTACCAGCAACCGGACAGTGAGCTTTG
Fn10(m6A9_s1)	Fn10-Fwd1	sense	TAGCCGTCTCGGATCTGTCACAGTGAGATATTAC
	Fn10_rev1(-)	antisense	GCCGTCTCGCCGCTAGGAGCATCCCAGCTGATC
Fn10(aMD4_s1)	Fn10-Fwd2	sense	AGCCGTCTCGGATCTAGCCCCGCAAGCAGCAAGC
	Fn10_rev2(-)	antisense	AGCCGTCTCGCCGCTACGGCCAGTGACAGCATAC
Fn10(m6A9_s2)	FnmP69-Fwd1	sense	AGGAAGGTTGATCGTGAGCCCCGCAAGCAGCAAGCC
	FnmP69-Rev1(-)	antisense	GTCCACCTCTCGATGTAAGGCCGCCAACGGCCAGTGAC
Fn10(aMD4_s2)	FnMD4-Fwd1	sense	GCACGAGGTGTGGAACTTGGATGGCAGCCCCGCAAGCAGCAAGC
	FnMD4-Rev1(-)	antisense	GTACGCCTGTTGAACTGCCTATAGCCTCCACGGCCAGTGACAGC
SIRPa(m6A9_s1)	mP69-Fwd34	sense	GGTGGACAGGAAGGTTGATCGTGGGCGGCAAAGAAGGCCACTTCCCCCG
	mP69-Rev34(-)	antisense	TCTCGATGTAAGGCCGCCAGCCTTGATTGTAGATTAATTCCC
SIRPa(m6A9_s2)	mP69-Fwd35	sense	GGTGGACAGGAAGGTTGATCGTGGGCGGCCCTGACACGGAG
	mP69-Rev35(-)	antisense	TCTCGATGTAAGGCCGCCAGCCGCTCCCTTTCCGGAACTTC
SIRPa(aMD4_s1)	MD4-Fwd28	sense	GCACGAGGTGTGGAACTTGGATGGCGGATCTAAAGAAGGCCACTTCCCCCG
	MD4-Rev28(-)	antisense	GTACGCCTGTTGAACTGCCTATAGCCGCCGCCTTGATTGTAGATTAATTCCC
SIRPa(aMD4_s2)	MD4-Fwd29	sense	CGTACGCCTGTTGAACTGCCTATAGCCGCCGCTCCCTTTCCGGAACTTC
	MD4-Rev29(-)	antisense	CACGAGGTGTGGAACTTGGATGGCGGATCTCCTGACACGGAG
CEA(m6A9_s1)	mP69-Fwd35	sense	TGGACAGGAAGGTTGATCGTGGGATCTCAACAAGCTACCCCAGGG
	mP69-Rev35(-)	antisense	CCTCTCGATGTAAGGCCGCCAACGGCCGCTTATTACATATCCTATAATTTG
CEA(m6A9_s2)	mP69-Fwd36	sense	TGGACAGGAAGGTTGATCGTGGGATCTCTTGTGAATGAAGAAGCAAC
	mP69-Rev36(-)	antisense	CCTCTCGATGTAAGGCCGCCAACGGCCGCTCTTTATGACGTGTAGGGTG
CEA(aMD4_s1)	MD4-Fwd30	sense	GCACGAGGTGTGGAACTTGGATGGCGGATCTCAACAAGCTACCCCAGGG
	MD4-Rev30(-)	antisense	GTACGCCTGTTGAACTGCCTATAGCCGCCGCTTATTACATATCCTATAATTTG
CEA(aMD4_s2)	MD4-Fwd31	sense	GCACGAGGTGTGGAACTTGGATGGCGGATCTCTTGTGAATGAAGAAGCAAC
	MD4-Rev31(-)	antisense	GTACGCCTGTTGAACTGCCTATAGCCGCCGCTCTTTATGACGTGTAGGGTG
VHH(m6A9_s1)	Nb-Fwd7	sense	TGGACAGGAAGGTTGATCGTAGGTCGCTCCTCCTACGAAGACTC
	Nb-Rev7(-)	antisense	CCTCTCGATGTAAGGCCGCCATCCCGAGGACATTCCTGCCACC
VHH(m6A9_s2)	Nb-Fwd9	sense	AGGTGGACAGGAAGGTTGATCGTGGGCTTCGAGTATTGGG
	Nb-Rev9(-)	antisense	CTCGATGTAAGGCCGCCAGCCCACGTTGACGTTACAATAG
VHH(aMD4_s1)	Nb-Fwd8	sense	ACGAGGTGTGGAACTTGGATGGAGGTCGCTCCTCCTACGAAGACTC
	Nb-Rev8(-)	antisense	GCGTACGCCTGTTGAACTGCCTATATCCAGCCGAGGACATTCCTG
VHH(aMD4_s2)	Nb-Fwd10	sense	ACGCACGAGGTGTGGAACTTGGATGGGGGCTTCGAGTATTGGGGCC
	Nb-Rev10(-)	antisense	ACGCCTGTTGAACTGCCTATAAGAGCCCACGTTGACGTTACAATAG
Fc(m6A9_s1)	mP69-Fwd4	sense	GGACAGGAAGGTTGATCGTGGGCGGAGACCCTGAGGTCAAGTTC
	mP69-Rev4(-)	antisense	ACCTCTCGATGTAAGGCCGCCAGCCGTGGCTCACGTCCACCACC
Fc(m6A9_s2)	mP69-Fwd2	sense	TGGACAGGAAGGTTGATCGTGGGCAACAGCACGTACCGtGTGG
	mP69-Rev2(-)	antisense	CCTCTCGATGTAAGGCCGCCAGCCGTACTGCTCCTCCCGCGGC
Fc(m6A9_s3)	mP69-Fwd3	sense	TGGACAGGAAGGTTGATCGTGGGCCCCATCGAGAAAACCATCTC
	mP69-Rev3(-)	antisense	CCTCTCGATGTAAGGCCGCCAGCCTGGGAGGGCTTTGTTGGAG
Fc(m6A9_s4)	mP69-Fwd32	sense	GGACAGGAAGGTTGATCGTGGGAGGGCAGCCCCGAGAACCAC
	mP69-Rev32(-)	antisense	ACCTCTCGATGTAAGGCCGCCACCCTTTGGCTTTGGAGATGG
Fc(m6A9_s5)	mP69-Fwd33	sense	TGGACAGGAAGGTTGATCGTGGGCGGCTCCTTCTTCCTCTACAG
	mP69-Rev33(-)	antisense	CCTCTCGATGTAAGGCCGCCACCCGGAGTCCAGCACGGGAGGC
Fc(m6A9_s6)	mP69-Fwd9	sense	GGACAGGAAGGTTGATCGTGGGCGGCCAGGTCAGCCTGACCTGC
	mP69-Rev9(-)	antisense	ACCTCTCGATGTAAGGCCGCCAGCCCTTGGTCAGCTCATCCCGG
Fc(m6A9_s7)	mP69-Fwd10	sense	GGACAGGAAGGTTGATCGTGGGCGGTGGGCAGCCGGAGAACAAC
	mP69-Rev10(-)	antisense	ACCTCTCGATGTAAGGCCGCCAGCCGCTCTCCCACTCCACGGCG
Fc(m6A9_s8)	mP69-Fwd11	sense	GGACAGGAAGGTTGATCGTGGGGGGGAACGTCTTCTCATGC
	mP69-Rev11(-)	antisense	ACCTCTCGATGTAAGGCCGCCACCCCTGCTGCCACCTGCTC
Fc(aMD4_s1)	MD4-Fwd4	sense	CGCACGAGGTGTGGAACTTGGATGGCGGAGACCCTGAGGTCAAGTTC
	MD4-Rev4(-)	antisense	TACGCCTGTTGAACTGCCTATAGCCTCCGTGGCTCACGTCCACCACC
Fc(aMD4_s2)	MD4-Fwd2	sense	GCACGAGGTGTGGAACTTGGATGGCAGCAACAGCACGTACCGTGTGG
	MD4-Rev2(-)	antisense	GTACGCCTGTTGAACTGCCTATAGCCTCCGTACTGCTCCTCCCGCGGC
Fc(aMD4_s3)	MD4-Fwd3	sense	GCACGAGGTGTGGAACTTGGATGGCAGCCCCATCGAGAAAACCATCTC
	MD4-Rev3(-)	antisense	GTACGCCTGTTGAACTGCCTATAGCCTCCTGGGAGGGCTTTGTTGGAG

Fc(aMD4_s4)	MD4-Fwd26	sense	ACGCACGAGGTGTGGAACTTGGATGGAGGGCAGCCCCGAGAACC
	MD4-Rev26(-)	antisense	ACGCCTGTTGAACTGCCTATAGCCCCCTTTGGCTTTGGAGATGG
Fc(aMD4_s5)	MD4-Fwd27	sense	ACGCACGAGGTGTGGAACTTGGATGGCGGCTCCTTCTTCCTCTAC
	MD4-Rev27(-)	antisense	ACGCCTGTTGAACTGCCTATAGCCCCCGGAGTCCAGCACGGGAG
Fc(aMD4_s6)	MD4-Fwd5	sense	CGCACGAGGTGTGGAACTTGGATGGCGGCCAGGTCAGCCTGACCTGC
	MD4-Rev5(-)	antisense	TACGCCTGTTGAACTGCCTATAGCCTCCCTTGGTCAGCTCATCCCGG
Fc(aMD4_s7)	MD4-Fwd6	sense	CGCACGAGGTGTGGAACTTGGATGGCGGTGGGCAGCCGGAGAACAAC
	MD4-Rev6(-)	antisense	TACGCCTGTTGAACTGCCTATAGCCTCCGCTCTCCCACTCCACGGCG
Fc(aMD4_s8)	MD4-Fwd7	sense	CGCACGAGGTGTGGAACTTGGATGGGGGGAACGTCTTCTCATGC
	MD4-Rev7(-)	antisense	TACGCCTGTTGAACTGCCTATATCCCCCCTGCTGCCACCTGCTC
Fc(m7_s8)	P7ds-Fwd8	sense	GCAAACTTACAGCTGGTATTGTGGATCGGGGAACGTCTTCTC
	P7ds-Rev8(-)	antisense	CAGGACAGCACGTTAGAATTGCAGCCGCTCCCCTGCTGCCAC
Fc(aMD5_s8)	MD5-Fwd9	sense	CAGACCTACAAAGCCTTTCCTGGATCGGGGAACGTCTTCTCATGC
	MD5-Rev9(-)	antisense	ATCCCAAGCATAGTACCAATAGCCGCTCCCCTGCTGCCACCTGCTC
Fc(A6-2f_s8)	A62f-Fwd7	sense	ATGAGTGGTACGTGGCTTATGGTTGTGGATCGGGGAACGTC
	A62f-Rev7(-)	antisense	CCAGCTCTTCAGGGAAAGAGGCGCAGCCGCTCCCCTGCTGCC
Fc(tkD5_s8)	tkD5-Fwd8	sense	AAAGTTCACCTTTTGGAGTTGTGGATCGGGGAACGTC
	tkD5-Rev8(-)	antisense	GGGGAAGCCTGGCGGTAGCAGCCGCTCCCCTGCTGCC
Fc(IB8_s8)	IB8-Fwd1	sense	GTGGTTGTATACCGTGGACGGCGGATCTAACGTCTTCTCATG
	IB8-Rev1(-)	antisense	CCGCTTCTTTTGACGAGGTATCCGCCGCTCTGCTGCCACCTG
Fc(A6-2f_s6)	A62f-Fwd6	sense	ATGAGTGGTACGTGGCTTATGGTTGTGGAGGCTCCCAGGTCAGC
	A62f-Rev6(-)	antisense	CCAGCTCTTCAGGGAAAGAGGCGCAGCCTCCGCTCTTGGTCAGC
hGH(m6A9_s1)	mP69-Fwd13	sense	TGGACAGGAAGGTTGATCGTGGGCAGCCCCCGGACTGGGC
	mP69-Rev13(-)	antisense	CCTCTCGATGTAAGGCCGCCAGCCATCTTCCAGCCTCTGC
hGH(m6A9_s2)	mP69-Fwd14	sense	TGGACAGGAAGGTTGATCGTGGGTGACGCACTACTCAAGAAC
	mP69-Rev14(-)	antisense	CCTCTCGATGTAAGGCCGCCAGCCGTTGTGTGAGTTTGTGTC
hGH(aMD4_s1)	MD4-Fwd40	sense	CGCACGAGGTGTGGAACTTGGACGGGGGCAGCCCCCGGACTG
	MD4-Rev40(-)	antisense	TACGCCTGTTGAACTGCCTATATCCGGAGCCATCTTCCAGC
hGH(aMD4_s2)	MD4-Fwd41	sense	CGCACGAGGTGTGGAACTTGGACGGGGGTGACGCACTACTC
	MD4-Rev41(-)	antisense	TACGCCTGTTGAACTGCCTATATCCGGAGCCGTTGTGTGAG
HSA(m6A9_s1)	mP69-Fwd5	sense	TGGACAGGAAGGTTGATCGTGGGCGGAGCTGAAAATTGTGACAAATC
	mP69-Rev5(-)	antisense	CCTCTCGATGTAAGGCCGCCAGCCCTCATCAGCAACACATGTTTTTG
HSA(m6A9_s2)	mP69-Fwd6	sense	TGGACAGGAAGGTTGATCGTGGGCGGTGATAAAGCTGCCTGCCTGTTG
	mP69-Rev6(-)	antisense	CCTCTCGATGTAAGGCCGCCAGCCAGCTTGGCAACATTCTGTAAAAG
HSA(m6A9_s3)	mP69-Fwd7	sense	TGGACAGGAAGGTTGATCGTGGGCGGAGATCCTCATGAATGCTATG
	mP69-Rev7(-)	antisense	CCTCTCGATGTAAGGCCGCCAGCCAGCGGCACAGCACTTCTCTAG
HSA(m6A9_s4)	mP69-Fwd8	sense	TGGACAGGAAGGTTGATCGTGGGCGGCGATAAGGAGACCTGCTTTG
	mP69-Rev8(-)	antisense	CCTCTCGATGTAAGGCCGCCAGCCAGCCTTGCAGCACTTCTCTAC
HSA(aMD4_s1)	MD4-Fwd8	sense	CGCACGAGGTGTGGAACTTGGATGGGTCAGCTGAAAATTGTGAC
	MD4-Rev8(-)	antisense	TACGCCTGTTGAACTGCCTATAGCCTGACTCATCAGCAACACATG
HSA(aMD4_s2)	MD4-Fwd9	sense	GCACGAGGTGTGGAACTTGGATGGCTCTGATAAAGCTGCCTGCCTG
	MD4-Rev9(-)	antisense	GTACGCCTGTTGAACTGCCTATAGCCAGCAGCTTGGCAACATTCTG
HSA(aMD4_s3)	MD4-Fwd10	sense	CACGAGGTGTGGAACTTGGATGGCTCAGATCCTCATGAATGCTATG
	MD4-Rev10(-)	antisense	CGTACGCCTGTTGAACTGCCTATAGCCTGCAGCGGCACAGCACTTC
HSA(aMD4_s4)	MD4-Fwd11	sense	GCACGAGGTGTGGAACTTGGATGGCGGCGATAAGGAGACCTGCTTTG
	MD4-Rev11(-)	antisense	GTACGCCTGTTGAACTGCCTATAGCCTCCAGCCTTGCAGCACTTCTC
RBP(m6A9_s1)	mP69-Fwd19	sense	TGGACAGGAAGGTTGATCGTGGGGAATAACTGGGACGTGTGCG
	mP69-Rev19(-)	antisense	CCTCTCGATGTAAGGCCGCCAGCCCAAAAGACGGACTCGGCCC
RBP(m6A9_s2)	mP69-Fwd20	sense	GGACAGGAAGGTTGATCGTGGGCTCCTTTCTCCAGAAAGGAAATG
	mP69-Rev20(-)	antisense	ACCTCTCGATGTAAGGCCGCCAGCCGGCTACGCCCCAGTACTTC
RBP(aMD4_s1)	MD4-Fwd42	sense	CGCACGAGGTGTGGAACTTGGACGGGGGGAATAACTGGGACGTG
	MD4-Rev42(-)	antisense	TACGCCTGTTGAACTGCCTATATCCGCCCAAAAGACGGACTCGGC
RBP(aMD4_s2)	MD4-Fwd43	sense	CGCACGAGGTGTGGAACTTGGACGGCTCCTTTCTCCAGAAAGG
	MD4-Rev43(-)	antisense	TACGCCTGTTGAACTGCCTATATCCGCCGGCTACGCCCCAGTAC
ALP(m6A9_s1)	mP69-Fwd18	sense	TGGACAGGAAGGTTGATCGTGGGAGGGGACGGCGCCCGGCCGGATG
	mP69-Rev18(-)	antisense	CCTCTCGATGTAAGGCCGCCAGCCTCCCTTGAGCACATAGCCTGG
ALP(aMD4_s1)	MD4-Fwd16	sense	GCACGAGGTGTGGAACTTGGATGGAGGGGACGGCGCCCGGC
	MD4-Rev16(-)	antisense	GTACGCCTGTTGAACTGCCTATAGCCTCCCTTGAGCACATAG
PA_s2_AAV1	PAins-Fwd	sense	GCCGAAGATGATGTGGTGGGATCTGACCCTGCGAC
	PAins-Rev(-)	antisense	ACCTGGCATGGCAACGCCGCCGCTGCTGCTCTGGAAATTG
aMD4_s2_AAV1	MD4-Fwd40	sense	GCGGCGGCTATAGGCAGTTCAACAGGCGCACGCACGAGGTGTGGAACTTGGATGGCG
	MD4-Rev40(-)	antisense	GATCCGCCATCCAAGTTCCACACCTCGTGCGTGCGCCTGTTGAACTGCCTATAGCCG
PA_s1_AAV2	pa-Fwd1	sense	CCGAAGATGATGTGGTGGGCACCACGCAGTCAAGGCTTC
	pa-Rev1(-)	antisense	CACCTGGCATGGCAACGCCTCCACTTGGAGTGTTTGTTC
PA_s2_AAV2	pa-Fwd2	sense	CCGAAGATGATGTGGTGGGACAAGCAGCTACCGCAGATG
	pa-Rev2(-)	antisense	CACCTGGCATGGCAACGCCGCCTCTCTGGAGGTTGGTAG
m6A9_s2_AAV2	mP69-Fwd25	sense	GGACAGGAAGGTTGATCGTGGGCACCACGCAGTCAAGGCTTC
	mP69-Rev25(-)	antisense	ACCTCTCGATGTAAGGCCGCCATCCACTTGGAGTGTTTGTTC

Supplementary Table S4. Primer sequences used for mutagenesis

Relevant construct	Primer name	Strand	Sequence(5'- 3')
pRC2-mi342_AgeI	pCR2mi342mut1+	sense	GATGCTGCGTACCGGtAACAACTTTACCTTCAG
	pCR2mi342mut1-	antisense	CTGAAGGTAAAGTTGTTaCCGGTACGCAGCATC
pRC2-mi342_NheI	pCR2mi342mut2+	sense	GCGGCAAAGTTTGCTagCTTCATCACACAGTAC
	pCR2mi342mut2-	antisense	GTACTGTGTGATGAAGctAGCAAACTTTGCCGC
